# Caregiver Perceptions of Children’s and Adolescents’ Psychosocial Functioning During the Stringent COVID-19 Lockdown Restrictions in Shanghai: Cross-sectional Study

**DOI:** 10.2196/43689

**Published:** 2023-02-07

**Authors:** Xu Liu, Jing Wu, Hongyang Yang, Fangjie Zhao, Yuchen Qin, Jiali Wu, Hongli Yan, Yan Xu, Lulu Zhang

**Affiliations:** 1 College of Health Service Second Military Medical University Shanghai China; 2 School of Nursing Second Military Medical University Shanghai China; 3 Xinhua Hospital Shanghai Jiao Tong University School of Medicine Shanghai China; 4 Institute of Hospital Development Strategy China Hospital Development Institute Shanghai Jiao Tong University Shanghai China

**Keywords:** COVID-19 exposure, psychosocial function, parenting, children and adolescents, China

## Abstract

**Background:**

The COVID-19 pandemic represents a global health crisis. The Shanghai municipal government in China implemented strict and comprehensive pandemic control strategies in the first half of 2022 to eliminate a wave of COVID-19 infection. The pandemic and the resulting government responses have led to abrupt changes to families’ daily lives, including the mental health of children and adolescents.

**Objective:**

The aim of this paper is to examine the impact of COVID-19 exposure and the stringent lockdown measures on the daily life and mental health of children and adolescents and to provide suggestions on maintaining their mental health when similar public health emergencies occur in the future.

**Methods:**

In this cross-sectional study, an anonymous survey was distributed online in May 1-15, 2022, in Shanghai. Individuals were eligible to participate if they were currently the caregiver of a child or adolescent (aged 4-17 years). Outcomes were psychosocial functioning of children and adolescents, as reported by parents, using the Pediatric Symptom Checklist-17. COVID-19 exposure and life changes were also reported. Multivariate logistic regression was used to analyze risk factors for poor psychosocial functioning.

**Results:**

In total, 2493 valid questionnaires were analyzed. The rate of positive scores on the global Pediatric Symptom Checklist-17 scale was 16.5% (n=411). Internalizing, attention, and externalizing problem subscale positivity rates were 17.3% (n=431), 10.9% (n=272), and 8.9% (n=221), respectively. Caregivers reported that 64.2% (n=1601) and 20.7% (n=516) of the children’s interactions with friends or peers and parents deteriorated, respectively. Compared with male caregivers, female caregivers were less likely to report psychosocial problems in children and adolescents (adjusted odds ratio [aOR] 0.68; 95% CI 0.53-0.88). Older children and those with lower COVID-19 Exposure and Family Impact Scales scores were less likely to have psychological problems (aOR 1.15; 95% CI 1.10-1.21). Compared with children with screen times <1 hour per day for recreation, those using screens for >3 hours had higher odds of psychological distress (aOR 2.09; 95% CI 1.47-1.97). Children who spent 1-2 hours exercising and had better interactions with friends or peers and parents showed a trend toward lower odds of psychological problems. Children and adolescents with worse sleep compared with preclosure were more likely to have psychological problems.

**Conclusions:**

The prevalence of psychosocial problems among children and adolescents is relatively high. Being young, having more COVID-19 exposure, and having more screen times (>3 h/day), less exercise time (<30 min), worse sleep, and deteriorated interactions with friends or peers and parents were risk factors for poor psychosocial functioning. It is necessary for governments, communities, schools, and families to take appropriate countermeasures to reduce the negative impact of the stringent control measures on caregivers’ parenting and psychosocial functioning of children and adolescents.

## Introduction

The COVID-19 pandemic represents a global health crisis. According to the latest data, as of June 7, 2022, there have been over 529 million confirmed cases of COVID-19 globally and over 6 million deaths [1]. Governments across the world have implemented several public health and social measures, such as mask wearing, school closures, international travel restrictions, and stay-at-home orders to prevent COVID-19 infection [1]. The unpredictability and uncertainty of the COVID-19 pandemic, the associated lockdowns and containment strategies, and the resulting economic disruption have led to abrupt and unpredictable changes to families’ daily lives and could increase the risk of mental health problems [2]. Previous studies showed that shielding behavior in both clinically extremely vulnerable and non–clinically extremely vulnerable populations during the first wave of the 2020 COVID-19 pandemic was associated with worse mental and physical well-being, suggesting that the adoption of such behavior may have resulted in avoidable detriments to physical and mental health [3].

In late February 2022, a wave of intense COVID-19 infection appeared in Shanghai, China. According to the Shanghai Municipal Health Commission, as of May 4, 2022, 593,336 cases have been identified, including 538,450 asymptomatic carriers [4]. To limit the spread of and subsequently eliminate COVID-19, the Shanghai municipal government implemented strict and comprehensive pandemic control strategies from March to May, including mask wearing, school and business closures, restrictions on domestic movement, public transport closures, and stay-at-home orders. According to the Public Health and Social Measures Severity Index issued by the World Health Organization, Shanghai’s policy was level 4 (of 5) in terms of severity [1]. The strict and comprehensive pandemic control strategies in Shanghai are designed to reduce the number of people infected and to allow for early diagnosis and appropriate treatment for severe COVID-19 cases [4]. Shanghai’s significant and stringent efforts against Omicron are essential for China as a whole to exit the pandemic situation.

Studies showed that the global pandemic and the resulting government responses have led to abrupt and unpredictable changes to damage to human health and creating burdens for families, health care systems, and societies [5-7]. As members of society, children and adolescents will not escape the repercussions of COVID-19, and inevitably, some children and families will experience these social costs differently. Efforts to contain the spread of COVID-19 have involved sudden, and often mandatory, physical distancing, thus removing many regular sources of social connection. School closures and quarantine orders may have contributed to a considerable proportion of the harms experienced by children [8] through a reduction in social contact with peers and teachers [9]. Many children have been unable to play or socialize outside the home. Social connections with classmates and peers were reduced, and adolescents especially suffer from a lack of social stimuli [10].

The mental health of children and adolescents during the COVID-19 crisis has attracted great attention [11]. In a systematic review of 36 studies from 11 countries, school closures and social lockdown during the first COVID-19 wave were associated with adverse mental health symptoms and negative health behaviors among children and adolescents [12]. Between 18% and 60% of children and adolescents scored above risk thresholds for distress, particularly anxiety and depressive symptoms [12]. Significant anxiety rates of 10%-19% and depressive symptom rates of 17%-39% have been estimated during the first COVID-19 wave [12]. In a longitudinal study, earlier internalization of symptoms increased the risk of clinically relevant depressive symptoms [13]. Another study suggested that 1 in every 4 young people globally are experiencing symptoms of clinical depression, while 1 in 5 are experiencing symptoms of clinical anxiety [8]. Assessments should be performed, and risk factors should be found as soon as possible to prevent serious mental problems from developing.

Longitudinal analysis indicated that more stringent policies and intense pandemics are associated with worse mental health [14]. This study aimed to examine the impact of COVID-19 exposure and the stringent lockdown measures on the daily life change and mental health of children and adolescents and provide suggestions on maintaining their mental health when similar public health emergencies occur in the future, leading to stringent lockdowns.

## Methods

### Recruitment

From May 1, 2022, to May 15, 2022, we conducted a municipal-wide web-based questionnaire survey in Shanghai, China. Individuals were eligible to participate if they were currently the caregivers of a child or adolescent (aged 4 to 17 years). Data were collected through the Wenjuanxing online questionnaire platform, on which only a fully completed questionnaire can be uploaded. An invitation letter, detailing the research aim and providing a QR code automatically generated by Wenjuanxing, was issued on widely used social platforms. Snowball sampling method and convenient sampling method were used [15]. Any individual interested in the study could use the QR code and fill in the questionnaire. In addition, we have taken a number of questionnaire quality-control measures, including the following: respondent as caregiver, who should be older than 20 years old; respondents with Shanghai internet protocol addresses; each social account can only fill in the questionnaire once; and respondents who took less than 2 minutes to complete the questionnaire were excluded.

### Ethical Considerations

This study was approved by the Ethics Committee of Xinhua Hospital Affiliated to Shanghai Jiaotong University School of Medicine (Approval Number: XHEC-C-2022-043). Appropriate ethical considerations were applied during all the stages of this study. Participation in the study was entirely voluntary. The survey questionnaire was distributed among respondents who provided their informed consent on the first page and clicking on the “agree” button after reading about the purpose of the survey. The questionnaire was anonymous to ensure the confidentiality and reliability of data.

### Measures

#### Demographic Characteristics of Caregivers and Children

Caregivers were asked to report the following information: age, gender, education level, living area, and number of children aged <18 years. In addition, the caregivers were asked to report the child’s age (4-18 years), gender, daily exposure time to screens for learning during school closures, daily exposure time to screens for recreation during school closures, amount of exercise per day during school closures, child’s sleep time compared to preclosure, child’s interactions with friends or peers compared to preclosure, child’s interactions with parents compared to preclosure, and child’s psychosocial symptoms.

#### COVID-19 Exposure and Family Impact Scales

The COVID-19 Exposure and Family Impact Scales (CEFIS) is a caregiver-report measure used to examine the degree to which families are exposed to potentially traumatic aspects of the COVID-19 pandemic, and the perceived impact on child, caregiver, and family functioning. It has been used to examine the impact of COVID-19 on various chronically ill pediatric populations [16,17], as well as on healthy children [18]. The CEFIS consists of the following three primary scales: Exposure, Impact, and Distress. Our study used the Exposure scale. The Exposure scale contains 25 “Yes/No” items that assess whether families have been exposed to COVID-19–related events such as lockdowns, school closures, changes in employment status, or the virus itself. When completing the CEFIS, residents are asked to think about the period from March 2022 to the present. “Yes” responses are summed to yield a total score, with higher scores indicating greater exposure to COVID-19 and related events.

#### Pediatric Symptom Checklist-17 Items

The Pediatric Symptom Checklist-17 (PSC-17) is one of the most frequently used general pediatric psychosocial screening instruments [19]. It is a caregiver-report measure of children’s psychosocial functioning. Parents rate the frequency of a variety of symptoms in their child (0=*never*, 1=*sometimes*, and 2=*often*). A screen can be considered “positive” based on total or domain scores. Total scores can be used to categorize a child as “at risk” or “not at risk” based on established cutoff scores. A score of ≥15 corresponds to at-risk status. On the three PSC-17 subscales, scores of ≥5 is for internalizing and ≥7 for externalizing; scores of attention ≥7 indicate risk. The PSC-17 has 3 subscales that specifically assess internalizing, externalizing, and attention-related problems.

### Statistical Analysis

Descriptive statistics were generated for respondent characteristics and CEFIS Exposure subscale scores. Caregiver and child age, as continuous variables, were presented as median with interquartile range, and were analyzed by the Wilcoxon rank sum test. Categorical variables are presented as numbers and percentages and were compared between poor psychosocial health (PSC-17 score ≥15) and good psychosocial health (PSC-17 score <15) groups using Pearson *χ*^2^ test or Fisher exact test. *P* values for trends were calculated using mental health as a binary categorical variable—event=PSC-17 score ≥15 (1), nonevent=PSC-17 score <15 (0)—and Pearson *χ2* trend test. To further examine potential risk and protective factors for mental health, univariate and multivariate logistic regression were performed. All respondent characteristic variables were included and adjusted in the multivariate logistic regression; unadjusted odds ratios, adjusted odds ratios (aORs), and their 95% CIs were calculated. All analyses were performed with SAS 9.4 (SAS Institute, Inc) and R statistical software (version 3.6.3; R Development Core Team). Statistical significance was set at *P*<.05, and all tests were 2-tailed.

## Results

### Descriptive Analysis of Participants

A total of 2540 valid questionnaires were returned. Questionnaires completed in <180 seconds were excluded, and 2493 valid questionnaires were finally analyzed. In total, 38.31% (955/2493) of the participants were male (Table 1), and 73.20% (n=1825) had 1 child (aged <18 years). In total, 52.55% (n=1310/2493) of the children were boys, and their average age was 10.99 (SD 4.18) years. Interactions with friends or peers and parents reportedly deteriorated in 64.22% (n=1601/2493) and 20.70% (n=516/2493) of the children, respectively.

**Figure 1:**
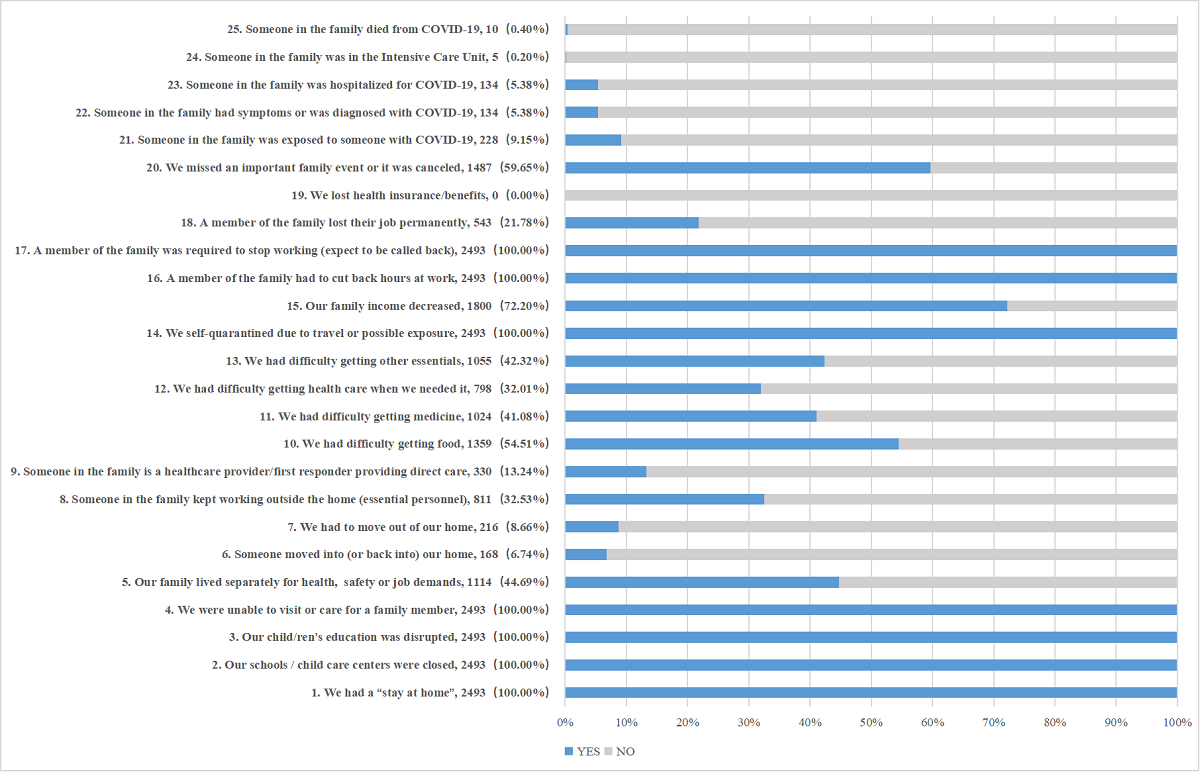
Individual COVID-19 Exposure and Family Impact Scales (CEFIS) item responses.

The mean CEFIS score was 11.5 (SD 2.49). The scores for individual CEFIS item are summarized in Figure 1.

**Table 1:** Participant demographic characteristics (N=2493).

Characteristics		Total (N=2493)	PSC-17^a^ score <15 (n=2082)	PSC-17 score ≥15 (n=411)	*P* value	*P* trend^b^
Caregiver’s age (years), median (IQR)		40 (36, 43)	40 (36, 43)	40 (36, 43)	.79	N/A^c^
**Caregiver’s gender, n (%)**					.008	N/A
	Male	955 (38.31)	773 (80.94)	182 (19.06)		
	Female	1538 (61.69)	1309 (85.11)	229 (14.89)		
**Education level, n (%)**					.49	.49
	High school and below	444 (17.81)	379 (85.36)	65 (14.64)		
	Junior college or BA	1532 (61.45)	1271 (82.96)	261 (17.04)		
	Postgraduate and above	517 (20.74)	432 (83.56)	85 (16.44)		
**Living area (square meters), n (%)**					.31	.23
	<30	890 (35.70)	730 (82.02)	160 (17.98)		
	30-50	653 (26.19)	553 (84.69)	100 (15.31)		
	>50	950 (38.11)	799 (84.11)	151 (15.89)		
**Number of children (<18 years old), n (%)**					.004	N/A
	1	1825 (73.20)	1548 (84.82)	277 (15.18)		
	≥2	668 (26.8)	534 (79.94)	134 (20.06)		
CEFIS^d^ score, median (IQR)		11 (10, 13)	11 (9, 13)	13 (11, 14)	<.001	N/A
Child age (years), median (IQR)		11 (8, 14)	11 (8, 15)	11 (7, 14)	.08	N/A
**Child age group, n (%)**					.046	.51
	4-6 years	473 (18.97)	402 (84.99)	71 (15.01)		
	7-12 years	1111 (44.56)	905 (81.46)	206 (18.54)		
	13-18 years	909 (36.47)	775 (85.26)	134 (14.74)		
**Child’s gender, n (%)**					.62	N/A
	Boy	1310 (52.55)	1089 (83.13)	221 (16.87)		
	Girl	1183 (47.45)	993 (83.94)	190 (16.06)		
**Child’s length of daily exposure to screens for learning during closure, n (%)**					.001	<.001
	≤4 hours	686 (27.52)	593 (86.44)	93 (13.56)		
	4-6 hours	637 (25.55)	543 (85.24)	94 (14.76)		
	6-8 hours	613 (24.59)	508 (82.87)	105 (17.13)		
	≥8 hours	557 (22.34)	438 (78.64)	119 (21.36)		
**Child’s length of daily exposure to screens for recreation during closure, n (%)**					<.001	<.001
	≤1 hour	634 (25.43)	556 (87.70)	78 (12.30)		
	1-2 hours	835 (33.49)	724 (86.71)	111 (13.29)		
	2-3 hours	414 (16.61)	350 (84.54)	64 (15.46)		
	≥3 hours	610 (24.47)	452 (74.10)	158 (25.90)		
**Child’s length of exercise per day during closure, n (%)**					<.001	<.001
	≤30 minutes	1370 (54.96)	1087 (79.34)	283 (20.66)		
	30 minutes to 1 hour	832 (33.37)	738 (88.70)	94 (11.30)		
	1 hour to 2 hours	219 (8.78)	200 (91.32)	19 (8.68)		
	≥2 hours	72 (2.89)	57 (79.17)	15 (20.83)		
**Child’s sleep compared to preclosure, n (%)**					<.001	<.001
	Much better	92 (3.69)	85 (92.39)	7 (7.61)		
	A little better	459 (18.41)	403 (87.80)	56 (12.20)		
	No change	1186 (47.57)	1065 (89.8)	121 (10.20)		
	A little worse	623 (24.99)	480 (77.05)	143 (22.95)		
	Much worse	133 (5.34)	49 (36.84)	84 (63.16)		
**Child’s interaction with friends or peers compared to preclosure, n (%)**					<.001	<.001
	Much better	45 (1.81)	40 (88.89)	5 (11.11)		
	A little better	161 (6.46)	145 (90.06)	16 (9.94)		
	No change	686 (27.52)	629 (91.69)	57 (8.31)		
	A little worse	880 (35.30)	751 (85.34)	129 (14.66)		
	Much worse	721 (28.91)	517 (71.71)	204 (28.29)		
**Child’s interaction with parents compared to preclosure, n (%)**					<.001	<.001
	Much better	131 (5.25)	121 (92.37)	10 (7.63)		
	A little better	564 (22.63)	495 (87.77)	69 (12.23)		
	No change	1282 (51.43)	1162 (90.64)	120 (9.36)		
	A little worse	398 (15.96)	267 (67.09)	131 (32.91)		
	Much worse	118 (4.73)	37 (31.36)	81 (68.64)		

^a^PSC-17: Pediatric Symptom Checklist-17 Items.

^b^Pearson χ2 trend test.

^c^N/A: not applicable.

^d^CEFIS: COVID-19 Exposure and Family Impact Scales.

### Bivariate Analysis of Psychosocial Functioning

As shown in Table 2, the proportion of positive screening scores on the global PSC-17 scale was 16.5% (411/2493). The internalizing, attention, and externalizing problem subscales had positive screening rates of 17.3% (431/2493), 10.9% (272/2493), and 8.9% (221/2493), respectively. Caregivers’ gender, number of children (younger than 18 years old), CEFIS score, child age, child’s length of daily exposure to screens for learning and recreation during closure, length of exercise per day, sleep, interaction with friends/peers and interaction with parents compared to pre-closure were significantly associated with psychosocial problems (Table 1).

**Table 2:** Caregiver reports of child mental health (N=2493).

Mental health	Positive screening scores, n (%)	Mean score (SD)
PSC-17^a^ total problems	411 (16.5)	8.23 (6.72)
PSC-17 attention problems	272 (10.9)	3.43 (2.51)
PSC-17 internalizing problems	431 (17.3)	2.24 (2.29)
PSC-17 externalizing problems	221 (8.9)	2.55 (2.62)

^a^PSC-17: Pediatric Symptom Checklist-17 Items.

### Logistic Regression Analysis

Multivariate logistic regression analysis showed that, compared with male caregivers, female caregivers were less likely to report psychosocial problems in children and adolescents (aOR 0.68; 95% CI 0.53-0.88). Older children and those with lower CEFIS scores were less likely to have psychological problems (aOR 1.15; 95% CI 1.10-1.21). Compared with children with screen times <1 hour per day for recreation, those using screens for >3 hours had higher odds of psychological distress (aOR 2.09; 95% CI 1.47-1.97). Compared with children who spent <0.5 hour exercising, those who spent 0.5-1 hour exercising had lower odds of psychological problems (aOR 0.70; 95% CI 0.52-0.94). Children who spent 1-2 hours exercising showed a trend toward lower odds of psychological problems. Children with worse sleep compared with preclosure and worse interactions with friends or peers and parents were more likely to have psychological problems (Table 3).

**Table 3:** Multivariate logistic regression analysis of characteristics associated with the mental health status of children and adolescents during the COVID-19–related school closures in Shanghai, China.

Characteristic		Unadjusted OR^a^ (95% CI)	*P* value	Adjusted OR (95% CI)	*P* value
Caregiver’s age		1.00 (0.98,1.01)	.65	1.01 (0.99,1.04)	.37
**Caregiver’s gender**					
	Male	Reference	N/A^b^	Reference	N/A
	Female	0.74 (0.60,0.92)	.007	0.68 (0.53,0.88)	.004
**Education level**					
	High school and below	Reference	N/A	Reference	N/A
	Junior College or BA	1.20 (0.89,1.61)	.23	1.14 (0.78,1.66)	.50
	Postgraduate and above	1.15 (0.81,1.63)	.44	1.09 (0.69,1.70)	.71
**Living area (square meters)**					
	<30	Reference	N/A	Reference	N/A
	30-50	0.83 (0.63,1.08)	.17	1.04 (0.76,1.42)	.82
	>50	0.86 (0.68,1.10)	.23	0.90 (0.68,1.20)	.49
**Number of children (<18 years old)**					
	1	Reference	N/A	Reference	N/A
	≥2	1.40 (1.12,1.76)	.004	1.52 (1.16,1.99)	.002
CEFIS^c^ score		1.24 (1.19,1.30)	<.001	1.15 (1.10,1.21)	<.001
Child age		0.98 (0.95,1.00)	.06	0.95 (0.91,0.99)	.01
**Child’s gender**					
	Boy	Reference	N/A	Reference	N/A
	Girl	0.94 (0.76,1.17)	.59	0.85 (0.66,1.08)	.18
**Child’s length of daily exposure to screens for learning during closure**					
	≤4 hours	Reference	N/A	Reference	N/A
	4-6 hours	1.10 (0.81,1.50)	.53	1.32 (0.92,1.90)	.13
	6-8 hours	1.32 (0.97,1.78)	.07	1.38 (0.94,2.01)	.10
	≥8 hours	1.73 (1.29,2.33)	<.001	1.20 (0.79,1.81)	.39
**Child’s length of daily exposure to screens for recreation during closure**					
	≤1 hour	Reference	N/A	Reference	N/A
	1-2 hours	1.09 (0.80,1.49)	.57	1.26 (0.89,1.79)	.19
	2-3 hours	1.30 (0.91,1.86)	.15	1.37 (0.91,2.07)	.13
	≥3 hours	2.49 (1.85,3.36)	<.001	2.09 (1.47,2.97)	<.001
**Child’s length of exercise per day during closure**					
	≤30 minutes	Reference	N/A	Reference	N/A
	30 minutes to 1 hour	0.49 (0.38,0.63)	<.001	0.70 (0.52,0.94)	.02
	1 hour to 2 hours	0.36 (0.22,0.59)	<.001	0.61 (0.35,1.06)	.08
	≥2 hours	1.01 (0.56,1.81)	.97	0.93 (0.45,1.92)	.84
**Child’s sleep compared to preclosure**					
	Much better	Reference	N/A	Reference	N/A
	A little better	1.69 (0.74,3.83)	.21	1.42 (0.58,3.50)	.45
	No change	1.38 (0.62,3.05)	.43	1.05 (0.44,2.54)	.91
	A little worse	3.62 (1.64,7.99)	.001	1.85 (0.76,4.48)	.18
	Much worse	20.82 (8.92,48.58)	<.001	5.32 (2.02,13.95)	<.001
**Child’s interaction with friends or partners compared to preclosure**					
	Much better, a little better, or no change	Reference	N/A	Reference	N/A
	A little worse	1.79 (1.33,2.42)	<.001	1.19 (0.85,1.66)	.31
	Much worse	4.12 (3.10,5.47)	<.001	1.70 (1.22,2.39)	.002
**Child’s interaction with parents compared to preclosure**					
	Much better	Reference	N/A	Reference	N/A
	A little better	1.69 (0.84,3.37)	.14	1.53 (0.72,3.25)	.27
	No change	1.25 (0.64,2.45)	.52	1.17 (0.56,2.44)	.68
	A little worse	5.94 (3.01,11.69)	<.001	4.03 (1.90,8.54)	<.001
	Much worse	26.48 (12.47,56.24)	<.001	10.51 (4.53,24.34)	<.001

^a^OR: odds ratio.

^b^N/A: not applicable.

^c^CEFIS: COVID-19 Exposure and Family Impact Scales.

## Discussion

### Principal Findings

This study examined the impact of the COVID-19 crisis on a sample of children and adolescents in Shanghai during the lockdown in the first half of 2022. This was the first study to assess the mental health of children and adolescents using the PSC-17 in China. Nearly one-sixth (16.5%, 411/2493) of the children and adolescents were reported to be with psychosocial function problems by their caregivers, which was similar to the results of a US study [20]. In another study, 18% of urban school-age children (5-11 years old) were at risk according to the total PSC-17 score, while 18% were at risk according to the PSC-17 internalizing symptoms subscale score [19]. Given the mental health impact on children and adolescents of COVID-19 exposure, measures should be taken to help families and parents optimize child health and development [21].

Psychological problems of children and adolescents varied cross different gender of reporting caregivers. Female caregivers reported less psychosocial problems in their children. The results were similar to those by Dave et al [22] but in contrast to the findings from Hagerman [23]. Dave et al [22] reported higher scores for externalizing behaviors, such as conduct problems and hyperactivity, among children (according to their fathers). Because of the stay-at-home policy, many fathers spent more time with their children, were involved in looking after them, and were more sensitive to externalizing behaviors than mothers, who were often the primary caregivers. The results showed that older children had fewer psychological problems. A possible reason for this might be that younger children were more susceptible to the crisis situation [24]. Those caregivers who had more than one child (younger than 18 years old) reported more psychological problems of their children. The reason might be that large families with more children living in cramped conditions might be under additional stress, including parenting-related stress, abuse, violence against children [25], and an increased risk of COVID-19 infection [26]. Government and community efforts are needed that target those at-risk households.

COVID-19 exposure was a risk factor for psychological problems of children and adolescents. The results of our study indicated severe COVID-19 exposure in our sample and increased risks of psychological problems. To eliminate COVID-19, stringent measures were implemented in Shanghai. Transport restrictions resulted in inconvenience in everyday life. Schools were closed, and children’s education was disrupted. Everyone, except essential workers, had to stay at home. Some families had difficulty obtaining essentials such as food, medicine, health care, and so on. Moreover, the shutdown had economic repercussions for many families, such as decreased income or unemployment. The infected families and those in which someone was serving as an essential worker outside the home had more difficulties with parenting and meeting the growth and developmental demands of their children. Government and community efforts are needed that target at-risk households. Children who are separated from their caregivers also require particular attention, including those infected with or suspected of being infected with COVID-19, those who are quarantined in local hospitals or medical observation centers, and those whose caregivers are infected with COVID-19 [27].

In this study, the rate of psychosocial problems increased with recreational screen time, consistent with other studies [28]. School closures and strict containment measures have led to more families relying on technology and digital solutions for their children’s learning, entertainment, and connection to the outside world [28,29]. Many children and adolescents are spending more time online, allowing for social interactions in some cases but also increasing the risk of cyberbullying, cybercrime, privacy infringements, and screen fatigue [10,30,31]. It has been proved that cyberchondria partially mediated the relationships between perceived severity of the COVID-19 pandemic and depression, anxiety, and stress in China [32]. As screen time increased, so too did sedentary behavior [33], which was associated with poor behavioral conduct or prosocial behavior and reduced sleep duration [34,35]. Worse sleep was a risk factor for the development of psychosocial problems in our study. Therefore, for children and adolescents, the time spent being sedentary should be limited, particularly the amount of recreational screen time [36]. Mutually agreed rules, limits, and parental monitoring of screen time were perceived as likely to be effective [37]. Interactive games and exercises can serve as a supplement for filling the time previously spent on screen.

Children and adolescents who spent 1-2 hours exercising showed a trend toward lower odds of psychological problems, and exercise for less than 30 minutes per day had a high probability of psychosocial problems. Psychosocial problems were linked to lower levels of physical activity [38-40]. Greater levels of physical activity are associated with improved physical fitness, bone health, cognitive outcomes, and mental health (reduced symptoms of depression) and less adiposity [36]. The World Health Organization recommends that children and adolescents perform at least 60 minutes of moderate-to-vigorous–intensity (mostly aerobic) physical activity per day. Vigorous-intensity aerobic activities, as well as those that strengthen muscle and bone, should be performed on at least 3 days a week [36]. High-quality physical education and supportive environments can promote physical and health literacy for long-lasting, healthy, active lifestyles [41]. Special attention should be paid to long-duration exercise. Previous studies had showed exercise durations of >3 hours were associated with worse mental health than exercising for 45 minutes [42]. In this study, exercise durations of 30 minutes to 1 hour were shown to be better for the psychosocial function of children and adolescents. Moreover, excessive exercise might cause fatigue and worsen mental health of children and adolescents [43].

In total, 27.9% of the caregivers reported that their interactions with children improved, which was significantly associated with lower positive screening scores. The stay-at-home policy might promote stronger relationships of children and adolescents with their parents [25] and has shown to improve their mental health [44]. Previous studies have reported that parent-child activities and exercises together can improve interactions between caregivers and children and amplifies the beneficial effects of sleep on mental health [45]. For example, mindfulness-promoting exercises (ie, yoga and tai chi) could be performed indoors by parents and children together [42]. In total, 64.2% (1601/2493) of the children and adolescents reported worse relationships with friends and classmates, and these children and adolescents were more likely to have poor mental health. There is an urgent need to increase the social interactions of children and adolescents during lockdown. Communities could redesign neighborhoods to provide children with spaces to play together and enjoy interactions [10]. Studies have shown that digitalized social contact can mitigate the potentially harmful effects of physical distancing on young people [9]. Specifically, active use of social media, for example, engaging in direct communication (ie, messaging) or posting directly on another person’s social media profile, can increase well-being and help maintain personal relationships. Time-limited and active use of social media for contact with peers might be beneficial for the mental health of children and adolescents.

### Limitations

This study had several limitations. First, its cross-sectional nature limits the ability to establish a causal relationship between risk factors and mental health. Furthermore, there were no preclosure PSC-17 data for Chinese children and adolescents, so we could not determine whether the psychosocial problems detected in the participants were intensified during COVID-19 exposure. Second, the data were collected online because of the COVID-19 pandemic, and caregivers whose lives were severely disrupted and had limited time online might have been less likely to finish the questionnaire. Third, data for the main variable of interest, psychosocial functioning, were obtained from caregivers of the children and adolescents, which may cause bias. When compared to self-report or direct observation of youth behaviors, caregiver report may not be as accurate.

### Conclusions

In this cross-sectional study conducted during a wave of COVID-19 infection in Shanghai, in which stringent responses were implemented, we found that the prevalence of psychosocial problems among children and adolescents was relatively high. Daily life of the children and adolescents changed a lot, including screen time, exercise, sleep, and interaction with parents and peers. Younger children, more COVID-19 exposure, more recreational screen time, and worse sleep were risk factors for poor psychosocial functioning. In particular, exercise durations of 1-2 hours and better interactions with friends or peers and parents benefit the psychosocial function of children and adolescents. The findings suggest that it is necessary for governments, communities, schools, and families to take appropriate countermeasures to reduce the impact of the lockdown measures on daily life and its subsequent influence on mental health of children and adolescents, which can be provided as suggestions on maintaining the mental health when similar public health emergencies occur in the future, leading to the stringent lockdown. In addition, it will be necessary to conduct longitudinal studies to clarify whether psychosocial problems are reduced, maintained, or intensified over time, as well as to identify subgroups of young people most at risk.

## Abbreviations

aOR: adjusted odds ratio
CEFIS: COVID-19 Exposure and Family Impact Scales
PSC-17: Pediatric Symptom Checklist-17 Items

## Data Availability

The original contributions presented in this study are included in the article. Further inquiries can be directed to the corresponding authors.
